# Neonatal and infant immunity for tuberculosis vaccine development: importance of age-matched animal models

**DOI:** 10.1242/dmm.045740

**Published:** 2020-09-15

**Authors:** Laylaa Ramos, Joan K. Lunney, Mercedes Gonzalez-Juarrero

**Affiliations:** 1 Mycobacteria Research Laboratories, Microbiology Immunology and Pathology Department, Colorado State University, 1682 Campus Delivery, Fort Collins, CO 80523, USA; 2 Animal Parasitic Diseases Laboratory, BARC, NEA, ARS, USDA Building 1040, Room 103, Beltsville, MD 20705, USA

**Keywords:** Immunity, Neonatal, Tuberculosis, Vaccines

## Abstract

Neonatal and infant immunity differs from that of adults in both the innate and adaptive arms, which are critical contributors to immune-mediated clearance of infection and memory responses elicited during vaccination. The tuberculosis (TB) research community has openly admitted to a vacuum of knowledge about neonatal and infant immune responses to *Mycobacterium tuberculosis* (Mtb) infection, especially in the functional and phenotypic attributes of memory T cell responses elicited by the only available vaccine for TB, the Bacillus Calmette–Guérin (BCG) vaccine. Although BCG vaccination has variable efficacy in preventing pulmonary TB during adolescence and adulthood, 80% of endemic TB countries still administer BCG at birth because it has a good safety profile and protects children from severe forms of TB. As such, new vaccines must work in conjunction with BCG at birth and, thus, it is essential to understand how BCG shapes the immune system during the first months of life. However, many aspects of the neonatal and infant immune response elicited by vaccination with BCG remain unknown, as only a handful of studies have followed BCG responses in infants. Furthermore, most animal models currently used to study TB vaccine candidates rely on adult-aged animals. This presents unique challenges when transitioning to human trials in neonates or infants. In this Review, we focus on vaccine development in the field of TB and compare the relative utility of animal models used thus far to study neonatal and infant immunity. We encourage the development of neonatal animal models for TB, especially the use of pigs.

## Abstract (precis)

**Summary:** Vaccine development for tuberculosis requires understanding of neonatal and infant immunity. Here, we compare the animal models of neonatal and infant immunity and highlight the potential of piglets as important models for vaccine development.

## Introduction

In 2018, the World Health Organization (WHO) received reports of 10 million new cases of tuberculosis (TB; Box 1), 11% of those occurring in children (WHO, 2020). TB in children is not considered a public health priority, and, as such, where the burden of TB is high in adults, reporting and diagnosing TB in children is deficient (WHO, 2015). Further, according to the WHO, in 2018, children accounted for 14% of deaths from TB without human immunodeficiency virus (HIV) and 13% of TB deaths in HIV-positive patients, suggesting a lack of access to prevention, diagnosis and treatment (WHO, 2020). The Bacillus Calmette–Guérin vaccine (BCG; Box 1) is currently the only vaccine licensed for this disease. Although BCG does not elicit immune responses that effectively prevent and control TB pathogenesis in adulthood (Box 2), it provides moderate protection from TB meningitis and miliary TB (Box 1) in children. For this reason, in endemic countries, BCG is recommended as part of neonatal vaccination programs. Moving forward, control of TB requires the development of an effective vaccination strategy that protects and controls TB from neonate to childhood and adulthood. Several strategies are under study, including replacing BCG with a new vaccine, or giving BCG at birth, followed by boosting with another dose of BCG or with a new vaccine later during infancy or adolescence (TBVI, 2020). The research community is working diligently towards this goal; however, as infants are not considered to cause significant transmission of TB (Tameris et al., 2013), the current pipeline of prospective TB vaccines has shifted from targeting disease prevention in infants towards adolescent and adult populations in which transmissible or active TB (Box 1) is highest (Ellis et al., 2015). The latter is also supported by modeling studies showing that vaccinating adolescents would decrease overall morbidity and mortality, and would increase the protection of infants compared to vaccinating infants alone with the same vaccine (WHO, 2015). Nonetheless, the clinical need and emphasis for neonatal and infant vaccine research remains of utmost importance because there are only limited treatment options for TB in children, and because children can act as a reservoir for future cases of TB in adolescents and adults. This is especially relevant for HIV-positive and other immunocompromised infants, for whom administration of BCG vaccine is contraindicated, leaving them vulnerable to TB and TB meningitis (Trunz et al., 2006). Since birth is likely to occur in a healthcare setting, vaccination against TB at this time would facilitate vaccination coverage and help decrease new infections in neonates and children (Kollmann et al., 2017). In 2019, the WHO Global TB program declared the diagnostics, prevention and treatment of TB in children part of the new WHO guidelines and road map for TB control and prevention (WHO, 2020).

**Box 1. TB terminology**

**Tuberculosis (TB):** an ancient communicable disease persistently afflicting millions of people around the world. TB is one of the top ten causes of death worldwide and the leading cause of death from a single infectious agent, ranking above human immunodeficiency virus (HIV)/acquired immune deficiency syndrome (AIDS). It typically affects the lungs (pulmonary TB) but can also affect other organs (extrapulmonary TB). In 2018, the World Health Organization (WHO) recorded more than 10 million new cases of TB.

***Mycobacterium tuberculosis* (Mtb):** the bacteria responsible for TB in humans and animals. The bacteria can spread in communities and households when people who are actively sick with TB expel bacteria into the air, for example, by coughing. About a quarter of the world's population is infected with Mtb and thus are at risk of developing active TB and spreading the infection further.

**Latent tuberculosis infection (LTBI):** LTBI is diagnosed in people infected with Mtb but not presenting clinical symptoms. Unlike active TB patients, LTBI individuals do not transmit the bacilli. LTBI is also defined as a state of persistent immune response to Mtb without clinically manifested evidence of active TB disease.

**Active TB:** found in TB patients with cavitary disease and clinical signs such as coughing, bloody sputum, night sweats and weight loss. During active TB, the pathogen can escape from its host and spread within the community via aerosols.

**Chemotherapy:** TB can be treated with antibiotics; however, treatment requires administration of several drugs over long periods of time, 6-8 months. This complex and lengthy regime means that many patients do not finish treatment. During the first 2 months, patients receive daily doses of isoniazid, rifapentine, ethambutol and pyrazinamide, followed by several months of treatment with isoniazid and rifapentine.

**Multidrug-resistant (MDR) TB:** lack of compliance with anti-TB treatment is one of the reasons for the emergence of MDR, and subsequently of extensively multidrug resistant (XDR), forms of TB. Treatment for these MDR and XDR TB infections requires over 2 years of administering very toxic cocktails of drugs.

**Bacillus** **Calmette–Guérin (BCG) vaccine:** first used in 1920, the BCG vaccine is the only vaccine available for TB. The vaccine has limited and variable efficacy in preventing TB in all populations, but endemic countries still administer it at birth because, when used in children, BCG can mitigate severe forms of TB, like TB meningitis and miliary disease. Non-endemic countries do not recommend use of BCG because of its interference with TB diagnostics.

**TB granulomas:** Mtb primarily infects macrophages; in the host, this develops into a granulomatous response, seen as infected macrophages surrounded by more macrophages and lymphoid cells, often T and B cells. Most macrophages within the granuloma develop a foamy aspect. The effect of the granulomatous response is to prevent the spread of bacilli in the tissue and to other organs.

**TB cavities:** usually form in the apices of the lungs or in the apical segments of the lower lobes.

**Tubercles:** nodules that contain caseous necrosis and form in the lungs as a result of TB infection.

**Miliary TB:** characterized by a wide dissemination of Mtb in the human body and by the tiny size of the lesions (1-5 mm).

**TB papules:** skin lesions measuring 1-3 cm in diameter that appear as friable, painful, erythematous-to-yellowish nodules. These lesions can lead to painful ulcers with fibrinous bases in the skin near bodily orifices. Papules may appear in middle-aged adults and seniors with advanced forms of lung, intestinal or genitourinary TB, or with severely impaired cellular immunity. Papules also appear after BCG vaccination or during the purified protein derivative (PPD) diagnostic test.

**Box 2. TB pathogenesis**

Mtb is an obligate human parasite causing what most often appears as a pulmonary disease. The mutual interaction between humans and Mtb has co-evolved for thousands of years and, yet, we are still searching for effective therapies and ways to control this pathogen. Mtb has survived in humans because it is capable of inducing pathologies that lead to necrosis and cavitation, and eventually spreading itself within the community via aerosol droplets. Conversely, humans have survived this pathogen because of a resilient and plastic immune system that can counterattack this pathogen. The latter may explain why the relationship between humans and Mtb bacilli is continually evolving and adapting to environmental conditions, and it also explains why, in humans, TB develops a wide spectrum of outcomes.

When Mtb enters the lungs via aerosol droplets, it encounters alveolar macrophage (AMs), cells that are permissive to Mtb infection and that act as the first replicative niche for the bacilli. Within AMs, the bacilli undergo rapid intracellular replication aimed at bursting the macrophage (necrosis) and thereby releasing progeny to the extracellular compartment to infect other macrophages. In other instances, the bacilli have limited capacity to replicate intracellularly and remain quiescent within the cell. In both cases, as the focal infection progresses, pro-inflammatory cytokines and chemokines, and recruitment of more macrophages and neutrophils along with innate lymphoid T cells, reshape the lung epithelium and eventually form a wall-like structure called a granuloma that contains the bacilli (Orme and Basaraba, 2014). Many AMs recruited to the site of infection develop a lipid-rich foamy morphology. Foamy cells eventually necrotize and so release their cargo, the intracellular Mtb and accumulated lipid droplets, into the focal site of infection. In all instances, control of bacterial growth and, importantly, control of necrosis, only happens if an army of adaptive IFNγ-producing Th1 cells infiltrates the site of infection or granulomas. Such control of bacilli growth and necrosis leads to a latent infection that, in this state, can persist for decades (Orme and Basaraba, 2014).

Primary TB, or the disease developed upon first infection with Mtb in a given host, is typically a systemic disease that lasts a few weeks. The bacilli reach several organs and can cause meningitis or disseminated (military) TB. After a few weeks, the host develops immune responses that are rarely sterilizing and are typically only capable of restraining the bacilli within granulomas. In this stage, called latent TB, the bacilli persist for decades. After 10-30 years, the bacilli may emerge from latency, leading to post-primary TB, producing cavities in the lung and permitting rapid proliferation of the bacilli, associated necrosis, cavities, cough and expulsion of bacilli in aerosol droplets (Hunter, 2011). Thus, while primary TB develops in a scenario of a naïve immune system, post-primary TB develops in the context of an existing immune response to TB.

To reduce the incidence of TB in children, the WHO recommends additional research to improve our understanding of the host-pathogen interaction of neonatal and infant immune responses to *Mycobacterium tuberculosis* (Mtb) (Box 1) infection (WHO, 2013). However, there are still many unknowns about the fundamentals of the neonatal immune system and its response to Mtb, which highlights the importance of using age-matched animal models to carry out these studies. Today, only a few studies on TB vaccine development for children have used neonatal and young animal models; thus, here we review the contributions of mice, guinea pigs, rabbits, calves, goats, pigs and non-human primates (NHPs) as TB animal models, with special emphasis on neonatal immunity studies. We compare this information to a recent report from our group and others on the immune response to BCG in young miniature pigs, emphasizing the potential of the pig as a viable model for neonatal TB vaccine development (Lee et al., 2004; Ramos et al., 2019).

## Neonatal immunity and responses to BCG

Overall, the immune responses of human infants are distinct and cannot be extrapolated from those of human adults or adult animal models (Sanchez-Schmitz and Levy, 2011; Saso and Kampmann, 2017). Neonates and infants have an actively changing immune system during the first 24 months of life, and show multiple innate and adaptive immune limitations that affect the ultimate outcome in immunity upon infection or vaccination. Immune defects in neonates span from cell migration to phagocytosis and bactericidal activity (Alexander-Miller, 2014). The innate response is limited at the level of Toll-like receptor (TLR) activation, dendritic cell (DC) maturation and breadth of DC subpopulations, including reduced plasmacytoid DC numbers and reduced MYD88 expression (Alexander-Miller, 2014). Neonates present with abundant circulating antimicrobial peptides early after birth and low levels of complement (Kollmann et al., 2017). The cytokines released by innate leukocytes have a different profile in newborns compared to adults (Kollmann et al., 2017). The few studies on neonatal immune cells show different and contradictory results; for example, tumor necrosis factor alpha (TNF-α; also known as TNF) levels in neonatal cells have been reported as significantly lower, equal or even higher than those in adult cells (Kollmann et al., 2009). Moreover, the current belief is that neonates can elicit a T-helper 1 (Th1) and T-helper 2 (Th2) primary T cell response (see Glossary, Box 3); however, the secondary immune response (Box 3) is thought to be biased towards Th2 (Zaghouani et al., 2009). The CD4^+^ T cells of newborns have a decreased capacity to produce interferon gamma (IFNγ), which might affect the magnitude of the Th1 response in newborns (Kollmann et al., 2017). This lack of Th1 secondary response in neonates (Zaghouani et al., 2009) is important for pediatric vaccines seeking to trigger Th1 responses to counter microbes and, therefore, should be studied further.

**Box 3. Glossary**

**Adjuvant:** a molecule, or complex of molecules, that enhances immune responses to antigens.

**CD1 proteins:** a family of glycoproteins present in various antigen-presenting cells. They are related to the major histocompatibility antigens and present non-peptide (lipid) antigens to T cells.

**Delayed-type hypersensitivity** **(DTH):** a unique type of cell-mediated immune response against an antigen that develops an inflammatory reaction if the host was previously exposed to that antigen. In the case of TB, when antigens derived from Mtb are injected in the skin, the site of injection may develop a papule as a result of positive DTH reaction with tissue necrosis. This reaction determines previous infection to Mtb and thus is used as a diagnostic tool.

**Interferon gamma (IFNγ) release assay (IGRA):** a whole-blood assay that measures levels of expression of IFNγ by blood leukocytes when exposed to TB antigens such as ESAT-6 and CFP-10 (also known as EsxB).

**Inversion of lymph nodes:** in some animals (pig, dolphins, hippopotamuses and rhinoceroses) the lymphoid cells (T and B cells) appear in an inverted distribution within the lymph nodes and, thus, when compared with most mammals, the lymph nodes are said to be inverted.

**Koch's postulates:** the criteria written by Koch in the late 19th century to establish a causal relationship between a causative microbe and a disease: (1) the microorganism must be found in abundance in all organisms suffering from the disease, but should not be found in healthy organisms; (2) the microorganism must be isolated from a diseased organism and grown in pure culture; (3) the cultured microorganism should cause disease when introduced into a healthy organism; and (4) the microorganism must be re-isolated from the inoculated, diseased experimental host and identified as being identical to the original specific causative agent.

**Non-invasive placenta:** a placenta that is loosely attached to the wall of the uterus.

**Peyer's patches:** small masses of lymphatic tissue found throughout the ileum region of the small intestine. These lymphoid nodules form an important part of the immune system by monitoring intestinal bacteria populations and preventing the growth of pathogenic bacteria in the intestines.

**Primary immune response:** develops when the host comes into first contact with an antigen.

**Secondary immune response:** immunity developed when the host is exposed to the same antigen a second, third and more times.

**Simian immunodeficiency virus (SIV):** an HIV-like virus that can infect monkeys and apes and can cause a disease similar to AIDS.

**Subunit vaccine:** a fragment of a pathogen (or antigen) with capacity to elicit protective immune responses against the pathogen of origin.

**T-helper 1** **(Th1) response:** characterized by CD4^+^ T cells expressing the cytokine IFNγ.

**T-helper 2** **(Th2) response:** characterized by CD4^+^ T cells expressing the cytokine interleukin-4 (IL-4).

**Trained immunity:** long-term boosting of innate immunity mechanisms in the host.

**Tuberculin skin test (TST):** the DTH reaction developed in the skin by patients previously exposed to Mtb and then exposed to Mtb a second time through intradermal injection. The TST is an important diagnostic tool used to determine previous exposure to Mtb.

**Fig. 1.**
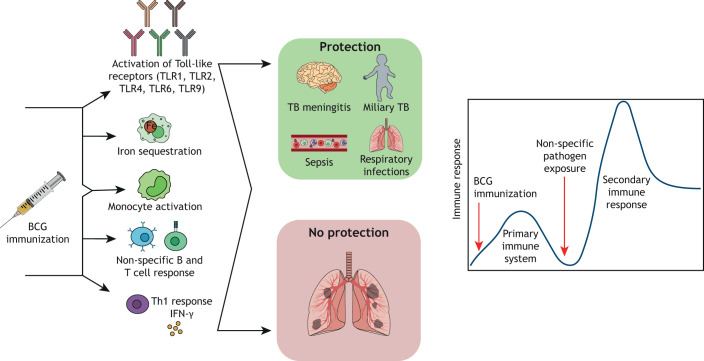
**Key features of BCG neonatal immune responses and protective characteristics.** Upon vaccination of a newborn, the BCG bacteria bind to and activate Toll-like receptors (TLR1, TLR2, TLR4, TLR6 and TLR9), leading to potent activation of innate immune responses. The antigens in the vaccine also activate monocytes and macrophages, and induce iron sequestration in these cells, which prevents pathogens from accessing this nutrient. BCG also elicits specific Th1, IFNγ-mediated, responses, along with non-specific B and T cell responses. The multiple layers of activation of innate and adaptive immunity elicited by BCG partly protect against TB. Moreover, BCG is now under study for its potential use as an adjuvant for other vaccines and for its capacity to stimulate trained innate immunity against common infant and childhood respiratory infections. The unique properties of BCG are also being studied in the context of the novel coronavirus SARS-CoV-2.

Experimental evidence from human and animal studies shows that BCG vaccination elicits non-specific effects in the immune system (Aaby et al., 2014), resulting in its activation in response to unrelated antigens (Kollmann et al., 2017; Netea and van Crevel, 2014). It has been known for a long time that BCG vaccination causes iron sequestration (Kochan et al., 1969), which depletes nutrients for microbes. BCG also promotes non-specific T and B cell responses, especially the expression of Th1 cytokines (Marchant et al., 1999). A unique feature of BCG compared to other vaccines is that BCG contains ligands for five distinct TLRs (TLR1, TLR2, TLR4, TLR6 and TLR9) (Randhawa and Hawn, 2008), leading to the speculation that the engagement of multiple TLRs contributes to its capacity to induce long-term boosting of innate immune mechanisms, an effect termed trained immunity (Netea and van Crevel, 2014) (Box 3; Fig. 1). For these reasons, BCG vaccination has acquired additional importance as an adjuvant (Box 3) to other vaccines administered early in life (Kollmann et al., 2017; Ota et al., 2002), and to boost children's immune responses against early microbial infections. In West Africa, for example, BCG has reduced neonatal mortality by more than 40% by preventing sepsis, respiratory infections and fever (Aaby et al., 2011). Interestingly, these non-specific and trained immunity properties of BCG have brought it to attention in the current SARS-CoV2 pandemic (Licciardi et al., 2020), prompting further discussions and research on harnessing its effects to fight this novel virus.

The efficacy of neonatal BCG vaccination (Fig. 1) has also been linked to its ability to polarize CD4^+^ Th1 responses early (Marchant et al., 1999); however, and for obvious reasons, there are few infant longitudinal studies that have followed the immune responses developed after neonatal BCG vaccination (Soares et al., 2008; Tena-Coki et al., 2010; Kagina et al., 2009). In these studies, the predominant T cell phenotypes were CD4^+^ effector T cells, which included the CD3^+^CD4^+^ and CD3^+^CD4^+^CD8a^+^ populations. Infants mounted a peak CD4^+^ T cell response 10 weeks after BCG vaccination (Soares et al., 2008); at the peak response, these CD4^+^ T cells expressed high levels of IFNγ, TNFα and interleukin (IL)-2. In a separate longitudinal study (Soares et al., 2013), central memory cells in participating infants ranged from 30% to 80%. As for expression of TNFα, infants appeared to maintain steady states ranging from 50% to 60% in CD4^+^ T cells from weeks 6 to 40, but these levels decreased at 1 year of age (Soares et al., 2013). These differences may be attributed to the different sampling timelines between studies. Two studies also describe CD8^+^ T cell activation, predominantly the effector phenotype (Tena-Coki et al., 2010; Soares et al., 2008), whereas most studies published so far report undetectable or low levels of IFNγ, TNFα and IL-2 production by CD8^+^ T cells (Kagina et al., 2009; Soares et al., 2008; Kagina et al., 2010). In summary, the exact mechanisms leading to the development of acquired immunity upon neonatal BCG vaccination and, most importantly, the mounting and duration of memory T cell responses remain poorly understood (Soares et al., 2013). Without clear information on how BCG induces the peak immune response of T cells and subsequent protective immune memory in neonates, progress in preventing TB will continue to stall.

All the above justify integrating effective vaccination against TB in a neonatal vaccination program. Below we briefly review the status of TB vaccine development for infants and discuss the available neonatal animal models to study immunity in the context of TB.

## TB vaccination development for pediatric use – a brief overview

There are currently 14 vaccine candidates in various phases of clinical trials (Table 1) to either prevent latent TB infection (LTBI; Box 1) or disease (WHO, 2020). These candidates may prove effective in prophylactic use, assisting chemotherapy (Box 1) or preventing disease relapse (Izzo, 2017). One of the most promising subunit vaccine (Box 3) candidates for children is the modified vaccinia Ankara virus-expressing antigen 85A (MVA85A), the first TB vaccine candidate to enter clinical trials in more than a decade (McShane and Williams, 2014; Tameris et al., 2013). MVA85A was designed as a booster to improve the protective efficacy of BCG (Tameris et al., 2013; Abubakar et al., 2013). A recent clinical trial enrolled nearly 3000 newborns vaccinated with BCG in South Africa to receive either MVA85A or a placebo (Tameris et al., 2013). The study concluded that MVA85A booster did not provide additional protection from TB, as no differences were observed between the number of infants who developed the disease between BCG with placebo and BCG with the MVA85A booster (Arnold, 2013). Nonetheless, MVA85A was further tested for safety and immunogenicity in a group of HIV-negative infants born to HIV-positive mothers as proof of principle. This study found that administration of MVA85A to HIV-exposed newborns did not affect BCG-induced immunogenicity and, as such, that other vaccine candidates could be administered safely to HIV-exposed newborns (Nemes et al., 2018).

**Table 1.**
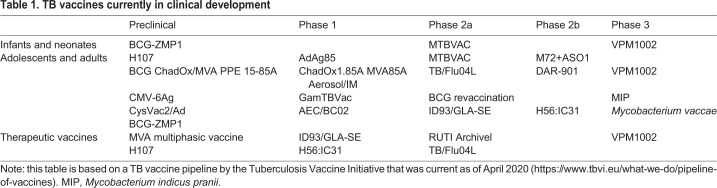
**TB vaccines currently in clinical development**

As of October 2019, the Tuberculosis Vaccine Initiative (TBVI, 2020) reports that out of the 14 vaccine candidates currently in various stages of clinical trials, three aim for administration to infants and neonates: BCG-ZMP1, MTBVAC and VPM1002 (Table 1). BCG-ZMP1, a recombinant BCG vaccine currently in preclinical testing, reduced bacterial loads in guinea pigs, increased IFNγ CD4^+^ T cell responses in cattle and was safe in immunocompromised mice when compared to BCG (Sander et al., 2015; Nieuwenhuizen and Kaufmann, 2018). The live-attenuated MTBVAC, currently in phase 2a of clinical trials and aimed at replacing BCG, has been tested in newborn mice and demonstrated safety, reduction of bacterial burden in the lungs and greater immune response compared to BCG (Marinova et al., 2017). In the phase 1a clinical trial, MTBVAC was safe and well tolerated by healthy adult volunteers without a BCG vaccination history (Marinova et al., 2017). In a dose-escalation trial, nine infants received MTBVAC and demonstrated durable antigen-specific CD4^+^ Th1 cells, promoting larger trials of MTBVAC in infants (Tameris et al., 2019). The recombinant BCG vaccine VPM1002, currently in phase 3 of clinical trials, was also aimed at replacing BCG. The VPM1002 demonstrated efficacy and safety over BCG in aerosol-challenged mice and was reported to be safe in newborn rabbits (Kaufmann et al., 2014). In a phase 2 clinical trial, 36 infants in South Africa received VPM1002, and, although overall found to be safe, the vaccine did not confer any differences in cytokine expression when compared to BCG (Loxton et al., 2017; Marinova et al., 2017). Other vaccine trials in infants, such as those of AERAS-402, a replication-deficient human adenovirus expressing multiple Mtb antigens, have proven safe and immunogenic. However, AERAS-402 did not induce an increased response of CD4^+^ T cells, even at increased doses (Tameris et al., 2015). This vaccine, along with MVA85A, was tested to compare the immune responses of infants to adults; the lower responses in infants highlighted differences between infants and adults that must be investigated further (Tameris et al., 2015). Thus far, these vaccine trials in infants have met the fundamental level of vaccine safety, but their immunogenicity profiles do not differ from that of BCG (TBVI, 2020). These trials would likely have benefited from more animal studies in the preclinical phase, and from the use of different and appropriately aged animal models, before proceeding to clinical trials in human infants.

## Neonatal animal models in TB vaccine development

Young infants in TB-endemic countries are at high risk of exposure to Mtb; when primary infection occurs in young children, the risk of dissemination to miliary disease and meningitis is significant (1-2%) (Marais and Schaaf, 2014). The younger the child, the higher the risk of TB dissemination, increasing the risk of serious sequelae or mortality for infected children (Marais and Schaaf, 2014). Undoubtedly, the most desirable option to control TB, and to avoid the establishment of Mtb infection, is rapid intervention very early in life and vaccination. In the case of primary TB (Box 2) in infants, questions remain about which type of immune mechanisms will stimulate protection against Mtb infection. How can researchers target these mechanisms in the context of an immature and naïve immune system that is continually evolving? How are these immune mechanisms going to establish short-, and even better, long-term immune memory? Most importantly, how can we answer these questions when, as discussed above, longitudinal studies of immune responses against Mtb infection or to vaccines in infants are scarce and logistically and ethically very difficult. The latter justifies the use of animal models. Although no single animal model can reproduce the full heterogeneity of outcomes of TB and granuloma (Box 1) in humans, there are several adult, and very few early life, animal models of TB helping to dissect the immune and pathobiological mechanisms of this disease. Ultimately, animal models offer an opportunity to test and understand the mechanisms of vaccine-mediated protection against Mtb. Although true vaccine efficacy in humans may be difficult to accurately estimate from animal models, a careful choice of parameters to be studied in each of the animal models available can yield insights into the likelihood of success of a vaccine candidate. Thus, we focus on the value of neonatal or early life animal models for TB candidate vaccine research.

## Mice

Many infectious disease studies involve the use of mice as immunological models and inbred strains are widely available. Additionally, mice are a low-cost model system, can be used in large numbers, and can be genetically engineered to express a preferred genotype best fit for a study design (Table 2) (Gurumurthy and Lloyd, 2019). Mice enable the study of immunity to TB in different genetic backgrounds. Several strains of mice (e.g. C57BL/6, BALB/c, C3HeB/FeJ) are used as models for preclinical TB vaccine studies (Henao-Tamayo et al., 2015a,b); each of these strains recapitulates unique features of human TB disease progression. Inbred strains such as C57BL/6 and BALB/c are susceptible to Mtb infection and develop pulmonary granulomas formed by large accumulations of macrophages, T cells and B cells, but do not develop necrotizing lesions (Gonzalez-Juarrero et al., 2001). Other inbred mouse strains, e.g. C3HeB/FeJ, DBA/2 and CBA/J, develop necrotizing responses to Mtb infection and C3HeB/FeJ and CBA mice develop TB cavities (Box 1) (Kramnik and Beamer, 2016). Notably, the necrotizing responses in these murine models reproduce those that precede cavity formation in humans and larger animal models, such as rabbits and NHPs (Kramnik and Beamer, 2016). It is also important to mention that not all human TB cases, such as those in an LTBI, present with necrotizing lesions. Therefore, it is essential to understand the disease and protection against Mtb infection in animal models with non-necrotizing and necrotizing types of disease. However, despite the fact that inbred adult mouse models can reproduce the heterogeneity of pulmonary TB observed in human patients (Kramnik and Beamer, 2016), the physiological and immunological distance between mice and humans cannot be ignored (Table 2) (Levast et al., 2013). The mouse model deserves credit for allowing researchers to understand the complexity of the immune system in an accessible and well-controlled system and for providing a customizable living vertebrate lung with the capacity for widespread experiments with definitive and reproducible outcomes (Cooper, 2015).

**Table 2.**
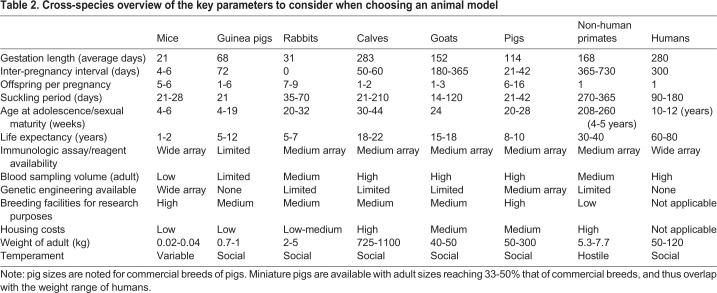
**Cross-species overview of the key parameters to consider when choosing an animal model**

Compared to human infants, newborn mice differ in cellular differentiation and anatomical structure and have an underdeveloped immune response (Hodgins and Shewen, 2012). In addition, murine developmental stages are much shorter than those of humans, with the suckling period only lasting 21-28 days, adolescence starting at 4-6 weeks and a life expectancy of 1-2 years (Perez-Cano et al., 2012). The species' short gestation of 18-21 days, and phylogenetic distance from humans, leaves the development of new infant vaccines short-handed if research is to rely on the mouse as the sole model system (Šinkora et al., 2002; Perez-Cano et al., 2012).

This is perhaps one of the reasons for the paucity of neonatal mouse studies in the field of TB research and development. Rahman and colleagues showed that 1-week-old (neonatal) and 4- to 6-week-old (infant) mice vaccinated with BCG mount a Th1 immune response, recapitulating the human infants' responses to BCG (Rahman and Fernandez, 2009; Vekemans et al., 2004). Kiros and colleagues came to the same conclusion after vaccinating mice with BCG at 5 and 7 days of age, challenging them with a range of BCG doses 16 weeks post-vaccination and determining that a low dose of BCG challenge elicits a predominant Th1 response (Kiros et al., 2010). Kamath and colleagues described for the first time that a novel subunit vaccine, Ag85B-ESAT-6, induces an adult-like multifunctional and protective response of CD4^+^ T cells in neonatal mice through the activation of DCs (Kamath et al., 2008). These neonatal mice produced similar levels of cytokines, such as IFNγ, TNFα, IL-17, IL-2 and IL-5, to those of the adult mice controls in the study. Moreover, using different adjuvants with the Ag85B-ESAT-6 vaccine triggered Th1 or Th2 response patterns (Kamath et al., 2008). This showed that adjuvants can modulate the neonatal immune response, stimulating a Th1 response, rather than the traditional Th2-biased CD4^+^ T cell activation observed in previous vaccine studies (Kamath et al., 2008). Although these neonatal mouse models resembled infants, most vaccine trials have used adult mice due to the short duration of the murine neonatal stage (Table 2). Therefore, for TB vaccine development, the mouse model should be used as a tool in identifying the potential mechanisms of protection, but should not be used to determine which neonatal or infant vaccine candidates move forward towards clinical trials (Cooper, 2015).

## Guinea pigs

Guinea pigs are highly sensitive to Mtb and can develop disease upon low-dose aerosol exposure to laboratory strains. To ensure that animals are treated humanely, they eventually require euthanasia after 100-150 days of Mtb infection (Williams et al., 2009). The disease in these animals resembles human TB, with granuloma formation with central necrosis surrounded by lymphocytes, inflammatory lesions, weight loss, and dissemination of bacilli to other sites of the lungs and other organs (Orme and Gonzalez-Juarrero, 2007; Clark et al., 2015; Lenaerts et al., 2007; Williams et al., 2009; Ordway et al., 2007; Grover et al., 2009). Guinea pigs are useful for demonstrating the progressive pathology of TB through its subacute, acute and chronic stages of infection (Ordway et al., 2008). In fact, guinea pigs were used by the pioneering TB bacteriologist Dr Robert Koch to initially study TB and to develop his four postulates (Box 3) of infectious disease etiology (Padilla-Carlin et al., 2008).

Guinea pigs are considered the gold standard model in vaccine testing, even though they are restricted to testing only vaccine efficacy, owing to a lack of commercially available immune reagents and assays (Table 2) to analyze the response at the cellular and molecular level. Thus, immunogenicity data for prospective vaccines must be derived from mouse studies, which is a problem because mice and guinea pig immune responses cannot be correlated to actual protection against infection with Mtb (Henao-Tamayo et al., 2015a; Clark et al., 2015). In addition to the lack of reagents, harvesting large blood samples from guinea pigs is problematic, limiting the immune response measurements during the course of a vaccine trial period (Tree et al., 2012). Nonetheless, compared to mice, guinea pigs have the advantage of larger size, closer hormonal and immunological similarities to humans, and, importantly, a wider range of pathological lesions following Mtb infection (Gupta and Katoch, 2005; Clark et al., 2015). Genetic studies of guinea pigs have shown immunological similarities to humans, e.g., homology between guinea pig and human CD1 proteins (Box 3) (Padilla-Carlin et al., 2008). The presence of CD1 in guinea pigs is important for vaccine studies, as these antigen-presenting surface proteins are not part of the murine immune system (Clark et al., 2015). The similarity in delayed-type hypersensitivity (DTH; Box 3) between guinea pigs and humans means that this model is useful for evaluating diagnostic skin test reagents (Clark et al., 2015). Additionally, guinea pigs acquire passive immunity *in utero*, but their guts do not absorb antibodies in colostrum or breast milk after birth (Pavia, 1986). Although the guinea pig genome has been fully sequenced, unlike mice, gene knockout, knock-in or transgenic guinea pig strains are not yet as available (Table 2) (Padilla-Carlin et al., 2008; Broad Institute, 2016). Further, pulmonary granulomas within Mtb-infected tissue rarely show liquification or cavitation, and guinea pigs do not develop LTBI (Padilla-Carlin et al., 2008).

Studies have shown a similarity between the human and guinea pig innate immune response and complement systems; however, a lack of immunological reagents for guinea pigs has prevented a full exploration of their response to TB infection and vaccination (Padilla-Carlin et al., 2008; Grover et al., 2009). Regardless, the guinea pig is considered the ‘gatekeeper’ for translating preclinical vaccine studies towards human clinical trials, even though their predictive value for humans remains unknown, because existing studies confirm that bacterial load reduction is the preferred vaccine efficacy readout in humans, rather than survival (Clark et al., 2015). Until reagents become readily available and optimized, studies of the immune response to vaccines and vaccine efficacy in guinea pigs will remain limited. A study in young guinea pigs found that they produce significantly lower levels of IgG in response to a pathogen compared to adults, a finding that parallels human neonatal and infant IgG production (Pichichero, 2014; Pilorz et al., 2005). Further, despite the fact that newborn guinea pigs possess a mature lympho-myeloid complex similar to that of human infants, to the best of our knowledge, studies in the field of TB using newborn guinea pigs as a model for disease or vaccine development do not yet exist (Gupta and Katoch, 2005).

## Rabbits

Rabbits have been used as primary models for infectious organisms such as HIV, human T-lymphotropic virus (HTLV1), human papillomavirus (HPV) and herpes simplex virus type 1 (HSV1) (Peng et al., 2015). The role of rabbits in TB research has been focused on distinguishing Mtb from *Mycobacterium* *bovis* infections (Manabe et al., 2003). When exposed to *M. bovis*, rabbits exhibit a chronic and progressive disease course with fibrous cavitary lesions and death upon inhaling fewer than 30 bacilli (Manabe et al., 2003). Additionally, the animals have demonstrated aerosol transmission with *M. bovis* (Griffin, 2000). For Mtb, rabbits can develop cavitary lesions and granulomas in the lungs, but the response depends on the Mtb strain and the rabbit genotype (Singh and Gupta, 2018). Hypervirulent strains of Mtb, such as W-Beijing, cause cavitary lesions, while CDC1551, a less virulent strain, results in LTBI (Esteves et al., 2018). Although rabbits can develop cavitary lesions, they are overall resistant to Mtb and clear the infection over time (Singh and Gupta, 2018; Manabe et al., 2003). Further, the animals require a high number of inhaled Mtb bacilli to produce one pulmonary lesion at 5 weeks (Manabe et al., 2003).

Although rabbits have contributed to our understanding of immunoglobulins and were used to develop the rabies vaccine (Esteves et al., 2018), their resistance to Mtb makes their role in TB vaccine development almost futile. However, because of their ability to arrest pulmonary tubercles (Box 1) early on, Dannenberg suggested including tubercle counts in vaccine studies using rabbits to avoid inconclusive vaccine trials (Dannenberg, 2010). Newborn rabbits were used to study the safety profile of VPM1002, demonstrating no influence on weight, dissemination to tissues, systemic toxicity or local intolerance reactions (Velmurugan et al., 2013). The immunogenicity profile to VPM1002 and pathogenic response to Mtb was not conducted in newborn rabbits, and instead the pathogenic response to VPM1002 challenged with Mtb was studied in adult mice (Velmurugan et al., 2013).

Rabbits are costlier animal models than rodents, but their medium size allows for increased blood sampling volume, along with more cells and tissue from a single animal (Table 2) (Esteves et al., 2018). Additionally, rabbits have a longer life span and an immune system more similar to that of humans than rodents (Esteves et al., 2018). However, the lack of Th1 cytokine reagents and other immunological assays limits vaccine studies (Singh and Gupta, 2018; Manabe et al., 2003). Further, with the exception of positive tuberculin skin tests (TSTs; Box 3), rabbits do not recapitulate TB clinical symptoms and shed Mtb in urine (Singh and Gupta, 2018; Manabe et al., 2008). Finally, transgenic rabbits, along with the rabbit genome, have only recently become available, opening the possibility for further studies of TB (Peng et al., 2015; Duranthon et al., 2012).

## NHPs

As the closest relative to humans, non-human primates (NHPs) are commonly used to model human disease based on their similar genome, physiology and immunology (Table 2) (Kaushal et al., 2012). Of particular relevance to TB research, NHPs demonstrate the spectrum of disease conditions such as LTBI, chronic infection and acute TB (Kaushal et al., 2012; Lin et al., 2014; Pena and Ho, 2016). More than half of experimentally infected macaques develop active disease, while 40% become latently infected (Myllymäki et al., 2015). Human-like LTBI does not present with clinical signs, but the antigen-specific immunological responses can be detected and measured by the TST or primate-specific IFNγ release assay (IGRA; Box 3) (Kaushal et al., 2012). The ability to study LTBI could aid in the development of antibiotic treatments and determine the role of granulomas in controlling Mtb (Fogel, 2015). Another major advantage of using NHPs is the potential to study TB/HIV co-infection, which affected 7.4-10% of new TB cases reported in 2018 (WHO, 2020). Macaques have been used to study the cellular, molecular and immunologic mechanisms of TB reactivation upon infection with simian immunodeficiency virus (SIV; Box 3) (Fogel, 2015). In 2013, Cepeda et al. demonstrated transient lesions, known as Ghon foci in human infants, in the lungs of neonatal rhesus macaques after aerosol Mtb infection (Cepeda et al., 2013), further confirming that NHPs adequately recapitulate human TB phenotypes. Despite these advantages, in addition to the ethical considerations for their use, the obvious limitation of studies in NHPs is the cost involved in their care, which limits the numbers of animals used for trials and the inevitable downstream effect on the statistical power of a given study (Table 2) (Kashangura et al., 2015). In addition, the two subspecies of macaques frequently used in research, rhesus and cynomolgus, mount variable responses to vaccines and Mtb challenge (Orme, 2015; Pena and Ho, 2016), and, depending on the method used to challenge the animals, researchers have observed different patterns of disease and have struggled to demonstrate protection by BCG vaccination (Orme, 2015). The consensus is to use NHPs as an endpoint model prior to clinical translation. However, despite their similarity to humans, the issues caused by their variable response to Mtb need to be resolved before NHPs can become the definitive model for TB research.

Not surprisingly, many similarities exist between NHP and human immune development, which means that neonatal NHPs can be useful models for neonatal and infant vaccine research. For instance, IgG levels are high at birth, decrease after a few months, and reach adult levels by the time macaques are 3-4 years old (Terao, 2009; Veazey et al., 2018; Shen et al., 2014). Upon intradermal BCG vaccination, infant macaques develop a papule (Box 1) and erythema at the site of injection (Wood et al., 2019), similar to humans. Further, infant macaques mount mycobacteria-specific CD4^+^ T cell responses that produce TNFα and IFNγ, but ultimately show variable efficacy of BCG vaccine upon Mtb challenge (Wood et al., 2019; Verreck et al., 2017). Different breeding backgrounds may be a confounding factor for this variable response to BCG (Verreck et al., 2017). For example, cynomolgus macaques develop high amounts of double-positive CD4^+^CD8^+^ T cells in the periphery, which further increase with age (Akari et al., 1997). Newborn macaques have been used to study BCG and its effects on HIV infection, as well as for TB vaccine trials. One study modeled the mother-to-child HIV transmission through breastfeeding by orally inoculating young macaques with SIV after intradermal BCG vaccination. The vaccinated animals showed elevated monocyte and T cell activation before and after SIV infection, while the influence of BCG on susceptibility to SIV infection was unclear (Wood et al., 2019). Another study addressed Mtb co-infection with SIV by evaluating the safety of a recombinant attenuated Mtb strain carrying SIV components through oral or intradermal administration. The vaccine's safety profile was successful, as the NHPs did not develop clinical symptoms or pathological changes, but immunological benefits, such as a rise in CD4^+^ T cells, were not observed (Jensen et al., 2012). In a later study by the same group, the authors reported persistent TB-specific immunity and low SIV-specific immunity after administration of a single oral dose of their attenuated Mtb-SIV vaccine to neonatal macaques (Jensen et al., 2013). In summary, the benefit of NHPs to model Mtb alone, or with SIV (HIV) co-infection, cannot be refuted. However, the use of neonatal NHP as preclinical vaccine models is limited by ethical concerns, single progeny per pregnancy and high cost.

## Cattle

The Mtb complex demonstrates host preferences but is not species specific. As such, cattle, which are naturally susceptible to *M. bovis* infections and develop bovine tuberculosis (bTB), have the potential to model Mtb strain infections as seen in humans (Villarreal-Ramos et al., 2018). A drawback is that bovine Mtb infections are less severe compared to *M. bovis* (Villarreal-Ramos et al., 2018)*.* Cattle mount a cellular immune response to Mtb, but do not develop pathological or bacterial loads (Waters et al., 2010). Nonetheless, when infected with *M. bovis*, cattle develop similar disease patterns to human Mtb, and outbred animals provide insight into the host genetics-mediated heterogeneity of disease patterns (Buddle et al., 2005). Cattle mount an IFNγ and antibody response to virulent *M. bovis* (Wedlock et al., 1999). The size of these animals permits collection of large volumes of blood and bronchoalveolar lavage fluid for various assays. However, their size also limits the number of animals available for study, or length of time they can be studied, owing to housing concerns (Table 2) (Guerra-Maupome et al., 2019).

The lack of vaccines against TB and urgent need for an effective vaccine is paralleled in bTB. The BCG response in cattle is similar to that in humans, meaning that it often fails to induce long-term protection (Buddle et al., 2005). Similar to humans, calves are immunocompetent at birth and demonstrate high levels of circulating natural killer (NK) cells (Buddle et al., 2005; Hamilton et al., 2016). Many BCG studies have been conducted in neonatal calves to investigate the effects of age at vaccination, BCG booster and adjuvants (Buddle et al., 2005). An *in vitro* study using peripheral blood mononuclear cells from young calves demonstrated that DCs mature as they take up BCG and further go on to produce Th1 cytokines and activate NK cells (Hamilton et al., 2016). Another study in 6- to 8-week-old calves found that aerosol BCG vaccination upregulates non-specific cytokine mRNA expression without changing monocyte phenotypes compared to unvaccinated animals (Guerra-Maupome et al., 2019). The use of neonatal calves has the potential to aid in vaccine development against both bovine and human TB; however, animal size and single offspring per pregnancy limit the use of cattle for longitudinal studies or as routine preclinical neonatal models (Table 2).

## Goats

Goats develop advanced human-like pulmonary TB and exhibit changes in body weight, gross pathology and bacterial load that can be measured to assess TB progression and vaccine efficacy. Goat models of *Mycobacterium* *caprae*, *M. bovis* and Mtb infection exist (Bezos et al., 2010, 2015; Gonzalez-Juarrero et al., 2013). After challenge, BCG-vaccinated kid goats showed reduced weight loss, smaller disseminated gross lesions in the lungs and lower bacterial load compared to unvaccinated goats (Vidal et al., 2017; Perez de Val et al., 2016). Goat kids vaccinated with BCG at 2 months of age that received an AdAg85A booster exhibited reduced pathology after Mtb challenge compared to BCG-only and unvaccinated control animals. Significant reductions in bacterial load were observed in both groups of vaccinated goats compared to unvaccinated controls; however, the reduction in bacterial load was more pronounced in AdAg85A-boosted animals. Importantly, antigen-specific IFNγ and humoral responses correlated with pathological and bacteriological results (Perez de Val et al., 2012), indicating that the immunological response in goats recapitulates that seen in conventional laboratory animal models and in the clinic. In summary, the goat may be used as a large animal model for TB research and is a viable alternative to the calf and NHP models, particularly because of relatively lower cost and easier maintenance. The key limitations are paucity of immune reagents as well as of facilities dedicated to breeding and housing these animals for research (Table 2). Furthermore, to our knowledge, there are no reports on neonatal goat BCG vaccination studies.

## Pigs

**Fig. 2.**
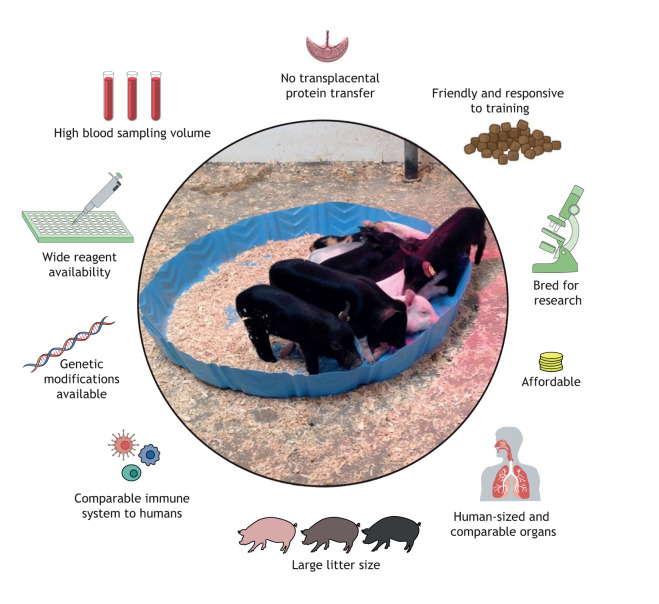
**Summary of the pig model characteristics and features that make it a suitable system for vaccine development.**

Domestic pigs have been compared to human subjects in a multitude of studies, as their anatomy, genetics, immune responses and physiology resemble those of humans (Fig. 2) (Rubic-Schneider et al., 2016; Swindle et al., 2012). This has resulted in increased use in research, and in the development of transgenic and gene-edited pig models to study human diseases such as Alzheimer's disease, cystic fibrosis and diabetes (Perleberg et al., 2018). Owing to functional and size similarities, pig-to-primate organ xenotransplantation models are well advanced as scientists plan for future pig-to-human xenotransplants (Ibrahim et al., 2006). There are many advantages to using pigs for human studies, such as their availability, size, ease of sampling, large litter size, friendly temperament and ethical acceptance (Table 2) (Meurens et al., 2012). The high cognitive abilities of pigs make them responsive to classical and operant conditioning, and thus they are easy to handle in laboratory studies (Gieling et al., 2011; Machado et al., 2017). Further, outbred and inbred lines of pigs, specifically developed for research purposes, are readily available (Meurens et al., 2012).

Pigs can be especially useful in human immunological studies as they can demonstrate the full scope of responses to vaccines or therapeutics as would be seen in humans (Meurens et al., 2012). Like the NHP and murine immune systems, the pig immune system is well characterized, and a wide range of resources for its study are available (Summerfield, 2009). The Swine Genome Sequencing Consortium has completed sequencing of the pig genome (Groenen, 2016; Groenen et al., 2012), which has led to further work in the pig immunome, revealing greater similarities to humans compared to mice (Dawson et al., 2013). About 50% of porcine immune studies focus on infectious disease (Summerfield, 2009). There are differences between the pig and human immune system: the inversion of lymph nodes (Box 3), two types of Peyer's patches (Box 3), and a passive immunity mechanism passed from a sow to her piglets (Meurens et al., 2012). The only way to transfer this passive immunity to a piglet is via the sow's colostrum and milk due to her non-invasive placenta (Box 3), which also prevents transplacental transfer of maternal proteins, including antibodies (Šinkora and Butler, 2016). Aside from these differences, the piglet immune system development is similar to that of humans and has been used extensively to study interactions between pathogens, e.g. influenza, and the immune system (Salmon et al., 2009; Meurens et al., 2012).

Pigs have a long gestational period of 114 days, allowing researchers to characterize the fetal immune system (Šinkora et al., 2002). Owing to the non-invasive placenta, fetuses can be maintained isolated and under sterile conditions *in utero*, enabling research to determine the mechanisms of the naïve immune system's interactions with microbes and to identify the immunological structural and functional changes during development (Šinkora et al., 2002). Using pigs to model the immune system could fill the gaps in our understanding of the response elicited by BCG vaccination in infants (Fig. 1) and improve vaccine efficacy outcomes.

Pigs have the same T cell populations as other jawed vertebrates: αβ T cells can focus on peptides presented by major histocompatibility complex molecules, while γδ T cells can recognize unprocessed antigens (Šinkora and Butler, 2016). Minimal to no double-positive T cells are observed before birth, and CD4^+^ T cells are the predominant phenotype in the spleen, mesenteric lymph nodes and umbilical blood. CD8^+^ T cells are less frequent, but increase with age (Šinkora et al., 2002). CD3^−^CD8^+^CD2^+^ NK cells amount to less than 5% of lymphoid cells in the spleen, mesenteric lymph nodes and umbilical blood in the second trimester of gestation (Šinkora et al., 2002). In adult pigs, NK cells can represent up to 15% of the lymphocyte population (Šinkora and Butler, 2009). Comparatively, human infants produce higher levels of NK cells early on, and these gradually decrease to reach adult levels at 5 years of age (Goenka and Kollmann, 2015). At birth, thymocyte populations in pigs resemble those of humans and mice, with the CD3^−^CD4^−^CD8^−^ thymocytes changing to double-positive CD4^+^CD8^+^ expression and finally to single-positive expression of either CD4^+^ or CD8^+^ cells (Murphy and Weaver, 2011). Outside the thymus, double-positive CD4^+^CD8^+^ expression on T cells has also been reported in both pig and NHP lymphoid tissues and blood (Gerner et al., 2015; Saalmuller et al., 1987). These double-positive cells were first described in humans (Blue et al., 1986), and have the capability of expressing memory markers while maintaining co-expression of CD4 and CD8 for 1 year in culture (Overgaard et al., 2015). Further, in ontogeny-development studies, co-expression of CD4 and CD8 on pig T cells continuously expands in the blood from birth to adulthood, and these double-positive cells proliferate upon antigenic stimulation and in tumor-like environments (Gerner et al., 2015).

Pigs, however, have a larger γδ T cell population and CD4^+^CD8^dull^ cells in blood and organs (Šinkora et al., 2002; Zuckermann, 1999). The adaptive immune response of piglets is considered naïve, and lymphocytes expand in response to the colonization of the gastrointestinal tract and exposure to pathogens (Šinkora and Butler, 2009; Butler et al., 2009). Similarly, human infants are considered naïve in their adaptive response and rely on their innate immune responses (PrabhuDas et al., 2011).

The use of pigs for TB research was first described in 2004. Lee, Molitor and colleagues monitored the γδ T cell response and IFNγ production in BCG-vaccinated infant pigs (Lee et al., 2004). Generally, αβ T cells are considered the most important T cell subtype in the immune response to TB; however, Lee at al.’s results indicate that this phenotype requires further study to identify the immunologic role of CD3^+^ T cells, the family that includes the γδ subtype (Lee et al., 2004; Ramos et al., 2019). In 2010, Cardona and his group challenged 1.5-month-old miniature pigs with the H37Rv Mtb strain and intervened with either the antibiotic isoniazid alone or with isoniazid combined with an Mtb fragment-based vaccine to study how TB affects pulmonary structure over 20 weeks (Gil et al., 2010). They found a strong Th1 response and weak humoral response to Mtb challenge. Isoniazid treatment alone decreased the total number of lung lesions, but increased the dissemination ratio, whereas isoniazid with the Mtb fragment-based vaccine boosted the Th1 and humoral responses, and increased the number of lung lesions, but reduced the dissemination ratio (Gil et al., 2010). The Th1 response to the Mtb fragment-based vaccine in these pigs further supports their use as vaccine models, as human infants predominantly mount a Th1 response to BCG (Marchant et al., 1999). Our group used an aerosol route to infect 2-month-old and 6-month-old miniature pigs, representing the respective human infancy and adolescence, with the hypervirulent strain Mtb HN878 (Ramos et al., 2017). We observed pathological changes, such as caseous necrosis and calcified lesions in the lungs and lymph nodes, recovered viable bacteria from both tissues, and demonstrated natural aerosol transmission from infected to uninfected pigs housed together (Ramos et al., 2017).

Further, our group studied the immune response of neonatal piglets that were vaccinated with BCG at birth and challenged with MtB at 20 weeks of life (Ramos et al., 2019). At 4 weeks of life, naïve T cells predominated, and by 6 weeks of life, effector CD4^+^ T cells had taken over. Current data for CD8^+^ T cell response to BCG are limited, but our study found that the numbers of CD8^+^ T cells remained stable after both BCG vaccination and Mtb challenge. Our study also showed unexpected patterns of intracellular and extracellular expression of IFNγ by T cells early in life, which need to be investigated further (Ramos et al., 2019). Overall, no significant differences were observed in T cell phenotypes in response to BCG or Mtb challenge between unvaccinated and vaccinated piglets. Our study presented unique findings on the high numbers of the double-positive CD4^+^CD8^+^ T-helper cell population in response to BCG and Mtb challenge. Whether this was induced by Mtb antigens, as found with other pathogens, or was an expected phenotype unique to pigs, remains unknown (Zuckermann, 1999). Further, BCG-vaccinated piglets had a higher number of activated monocytes at 4 and 6 weeks of life compared to unvaccinated piglets, and this trend continued as the piglets aged and after they received the Mtb challenge (Ramos et al., 2019). This active monocyte population could have been undergoing epigenetic reprogramming and trained immunity, a process that BCG induces in humans (Byrne et al., 2020). However, affirming this will require further study (Marinova et al., 2017). Our work demonstrated parallels between piglets and human infants vaccinated with BCG, indicating that future vaccine studies could benefit from using neonatal piglets prior to initiating clinical trials (Fig. 2) (Ramos et al., 2019).

Overall, these studies prove the potential pigs have for modeling TB. Upon Mtb infection, pigs develop similar pathological changes in the lung tissue and parenchyma to humans, including pulmonary tubercles with caseous necrosis, followed by liquefaction and cavity formation (Ramos et al., 2019; Helke et al., 2006), and mount immune responses that recapitulate the human response to Mtb infection and BCG vaccination (Gil et al., 2010; Lee et al., 2004; Ramos et al., 2019; Ramos et al., 2017). This, combined with their size and anatomy (Table 2), affirms that neonatal pigs are suitable models for neonatal and infant human TB vaccine research.

## Conclusions

The research discussed in this article underscores the relevance of continued research into the mechanisms of the neonatal immune response, both in the context of BCG and in novel pediatric vaccine development for TB control. Moving forward, neonatal and early-life animal models need to become essential tools to advance research in the area of a neonate's mechanisms of protection from Mtb microbial invasion and pathogen resistance. Ultimately, knowledge obtained from such studies should inform the development of more effective and safe pediatric TB vaccines.

Animal models are crucial for vaccine research. Whereas aging immunity research has centered on developing aging animal models (Pawelec and Larbi, 2008), the neonatal-infant immunity field has fallen behind. This is particularly jarring as the immunological discrepancies between adults and infants are well known (Marchant and Goldman, 2005). Animal models have provided deep insight into TB pathology and the elicited immune response; however, for more than 100 years, the research community has been hindered from developing effective therapies or vaccines to prevent TB. We should also bear in mind that research will always be limited by the fact that there is no perfect animal model to study TB, nor is there a perfect model that replicates human BCG responses. It is most likely that we never will have a perfect animal model. In fact, we will likely need to continue using multiple models to thoroughly decipher the responses to Mtb, as the course of TB in humans is an ever-evolving spectrum that varies widely (Box 2). We may also need several models to fully understand the immune mechanisms of protection elicited by TB vaccines. Here, we have discussed the lack of age-matched animal models in preclinical studies of TB vaccine development, especially neonatal-infant studies, and recognized the contributions of mice, guinea pigs, rabbits, cattle, goats, NHPs and pigs to TB and vaccine research efforts. For some of these animal models, studies will remain limited due to restrictions inherent to these animals (Table 2). Additionally, except for the murine models, reagents such as monoclonal antibodies and expressed immune proteins are not widely available for most of these animals, and researchers must rely on other laboratories or veterinary immunology consortia for their production.

Studies in neonatal immunology and TB are few. Here, we have highlighted the pig as a realistic animal model for TB research and as a specific age-matched animal to model vaccine development for TB (Fig. 2). Trials in neonatal piglets, with their resemblance to human infants, could provide insight into the BCG-induced T cell responses and should be developed further to test other vaccine candidates (Fig. 2) (Meurens et al., 2012; Butler et al., 2009; Ramos et al., 2019). Future preclinical vaccine studies can benefit from using neonatal and young animal models, and we suggest using pigs for TB vaccine development as an auxiliary to the limited immunological information in human neonates. As an animal model, pigs could ultimately provide more stable results for vaccine immunogenicity and efficacy trials, assuring better data before progressing to human clinical trials.
